# Associations between rapid auditory processing of speech sounds and specific verbal communication skills in autism

**DOI:** 10.3389/fpsyg.2023.1223250

**Published:** 2023-08-17

**Authors:** Carly Demopoulos, Sara A. Skiba, Brandon E. Kopald, Nitin Bangera, Kim Paulson, Jeffrey David Lewine

**Affiliations:** ^1^Department of Psychiatry and Behavioral Sciences, University of California, San Francisco, San Francisco, CA, United States; ^2^Department of Radiology and Biomedical Imaging, University of California, San Francisco, San Francisco, CA, United States; ^3^Ape Cognition and Conservation Initiative (Ape Initiative), Des Moines, IA, United States; ^4^Department of Neurology, University of California, San Francisco, San Francisco, CA, United States; ^5^Mind Research Network, Albuquerque, NM, United States; ^6^Departments of Psychology and Neurology, University of New Mexico, Albuquerque, NM, United States; ^7^Center for Advanced Diagnostics, Evaluation and Therapeutics, CADET-NM, Albuquerque, NM, United States

**Keywords:** autism spectrum disorder, magnetoencephalography, auditory processing, communication, speech, language

## Abstract

**Introduction:**

The ability to rapidly process speech sounds is integral not only for processing other’s speech, but also for auditory processing of one’s own speech, which allows for maintenance of speech accuracy. Deficits in rapid auditory processing have been demonstrated in autistic individuals, particularly those with language impairment. We examined rapid auditory processing for speech sounds in relation to performance on a battery of verbal communication measures to determine which aspects of verbal communication were associated with cortical auditory processing in a sample of individuals with autism.

**Methods:**

Participants were 57 children and adolescents (40 male and 17 female) ages 5–18 who were diagnosed with an Autism Spectrum Disorder (ASD). Rapid auditory processing of speech sounds was measured via a magnetoencephalographic (MEG) index of the quality of the auditory evoked response to the second of two differing speech sounds (“Ga” / “Da”) presented in rapid succession. Verbal communication abilities were assessed on standardized clinical measures of overall expressive and receptive language, vocabulary, articulation, and phonological processing. Associations between cortical measures of left- and right-hemisphere rapid auditory processing and verbal communication measures were examined.

**Results:**

Rapid auditory processing of speech sounds was significantly associated with speech articulation bilaterally (*r* = 0.463, *p* = 0.001 for left hemisphere and *r* = 0.328, *p* = 0.020 for right hemisphere). In addition, rapid auditory processing in the left hemisphere was significantly associated with overall expressive language abilities (*r* = 0.354, *p* = 0.013); expressive (*r* = 0.384, *p* = 0.005) vocabulary; and phonological memory (*r* = 0.325, *p* = 0.024). Phonological memory was found to mediate the relationship between rapid cortical processing and receptive language.

**Discussion:**

These results demonstrate that impaired rapid auditory processing for speech sounds is associated with dysfunction in verbal communication in ASD. The data also indicate that intact rapid auditory processing may be necessary for even basic communication skills that support speech production, such as phonological memory and articulatory control.

## Introduction

Abnormalities in auditory processing are a well-documented finding in studies of Autism Spectrum Disorder (ASD), and present at both the cortical and subcortical level (Klin, 1993; Bonnel et al., 2003; Alcántara et al., 2004; Oram Cardy et al., 2004; Heaton, 2005; O’Riordan and Passetti, 2006; Tas et al., 2007; Russo et al., 2009; Roberts et al., 2010, 2013; DePape et al., 2012; Mayer et al., 2014; Tomchek et al., 2014; Kargas et al., 2015; Stewart et al., 2015; Demopoulos and Lewine, 2016). Prior research has also linked auditory processing abnormalities to communication impairment in ASD (Gage et al., 2003; Oram Cardy et al., 2005; Roberts et al., 2008, 2011, 2019; Schmidt et al., 2009; Edgar et al., 2013, 2014; Port et al., 2015; Berman et al., 2016; Demopoulos et al., 2017, 2023; Matsuzaki et al., 2019, 2020). Magnetoencephalography (MEG) has been used to quantify delays in cortical response to basic auditory stimuli in autism (Demopoulos et al., 2015, 2017; Roberts et al., 2019, 2021). Studies that have examined the relation of these cortical auditory response abnormalities to language skills have reported associations between delayed peak latency and lower Wechsler Intelligence Scale for Children-Fourth Edition (WISC-IV; Wechsler, 2003) Verbal Comprehension Index scores (Demopoulos et al., 2017) as well as absent responses in autistic children classified as language-impaired based on Clinical Evaluation of Language Fundamentals-4th Edition (CELF-4; Semel et al., 2003) scores (Edgar et al., 2014). Further, there is evidence that latency delays are apparent early in development, as Riva et al. (2018) reported delayed MMF latency to complex tone stimuli at 12 months of age in a sample of infants at risk for ASD and language impairment, respectively, compared to controls. Moreover, these latencies were associated with expressive vocabulary scores on the Language Development Survey (Rescorla and Alley, 2001) measured at age 20 months in the combined sample.

These auditory latency delays and absent responses have been hypothesized to impact the ability for the brain to process auditory information at the rapid pace necessary for the comprehension and production of speech. This is supported by research demonstrating an association between higher auditory gap detection thresholds in ASD (Bhatara et al., 2013), which have been associated with weaker phonological processing measured via the Comprehensive Test of Phonological Processing (CTOPP; Rashotte et al., 1999; Foss-Feig et al., 2017). Paradigms involving two auditory stimuli presented in rapid succession (rapid auditory processing paradigms) have been applied to investigate this relationship. Indeed, rapid cortical auditory processing impairments were identified in individuals with ASD who demonstrated deficits in overall language abilities, classified by Below Average performance on the CELF-4, and/or phonological processing, defined by Below Average performance on the CTOPP (Oram-Cardy et al., 2005). Recent work suggests that this association may have been driven by phonological processing impairment rather than overall language abilities, as Demopoulos et al. (2023) failed to identify a direct relationship between rapid auditory processing for of puretone sounds and overall language abilities in children and adolescents with ASD. Instead, phonological processing ability mediated the association between rapid processing and language, whereas direct associations were only identified between rapid processing and the component skills that support language, including phonological awareness, speech articulation, and expressive and receptive vocabulary. Notably, both of these studies examining relations between rapid auditory processing and language skills employed basic auditory stimuli (i.e., clicks or puretone sounds, respectively). No study to date has examined associations between rapid cortical processing of speech sounds and overall language abilities in individuals with autism, yet prior research has demonstrated a differential cortical response to speech relative to more basic auditory stimuli such as that utilized in Oram-Cardy et al. (2005) and Demopoulos et al. (2023). For example, longer response latencies to speech stimuli relative to tone bursts has been demonstrated in both neurotypical and autistic children (Kamita et al., 2021).

Indeed, differences in cortical auditory processing of speech stimuli have been repeatedly identified in autism study samples. Specifically, longer MMF latencies to vowel sound stimuli were associated with lower scores on the CELF-4 in individuals with ASD (Roberts et al., 2011; Berman et al., 2016). In minimally verbal and nonverbal children with ASD, Matsuzaki et al. (2019) found that MMF latencies to vowel stimuli were delayed in both hemispheres, as compared to verbal children with ASD and typically developing children. Notably, parent-reported communication skills, including expressive, receptive, and written communication, assessed via the Communication Domain score of the Vineland Adaptive Behavior Scale, Second or Third editions (Sparrow et al., 2005, 2016), accounted for significant variance in MMF latency for all groups. Thus, the weight of the evidence suggests that slower cortical auditory response behavior is associated with verbal communication impairments in individuals with autism.

Previous work suggests that this slower cortical auditory response may impact verbal communication abilities by way of impaired processing for rapidly presented auditory information. Specifically, rapid processing of basic sounds (Oram-Cardy et al., 2005; Demopoulos et al., 2023), was directly associated with phonological processing, and indirectly associated with overall language skills via a mediating effect of phonological processing (Demopoulos et al., 2023). It remains unclear whether rapid processing of speech sounds, an integral component of phonological awareness, is directly associated with overall language abilities. To address this gap in the extant literature, we evaluated associations between rapid cortical auditory processing of speech sounds and performance on measures of both overall expressive and receptive language skills, as well as performance on measures of more basic components of language, including receptive and expressive vocabulary, articulation, phonological awareness, phonological memory, and rapid naming in children and adolescents with ASD. Given the evidence of a mediating effect of phonological awareness on the relation between rapid auditory processing of puretones and language (Demopoulos et al., 2023), we hypothesized that impaired rapid cortical auditory processing of speech sounds would be directly associated with overall language abilities as well as the basic skills in verbal communication that support language. Identifying the cortical processes that support the functional component skills of language in children with autism is critical to understanding the barriers to development of verbal communication skills for some individuals with ASD and potentially for other neurodevelopmental disorders as well.

## Methods

### Participants

MEG scans were attempted for 66 participants as part of a study on the neurobiology of language impairment in ASD (R01HD051747-01A1). Scans were discontinued prior to administration of the rapid speech sound processing task reported in this study for five participants due to difficulty remaining still or the participant’s desire to discontinue. Four additional participants completed the scan but their data did not meet quality standards for processing due to too few artifact-free trials. Participants with usable data were 57 children (40 males, 17 females) ages 5–18 (*M* = 10.49, SD = 3.14) with a DSM-IV-TR diagnosis of an autism spectrum disorder (ASD), including Autistic Disorder (*N* = 38), Asperger’s Syndrome (*N* = 14), or Pervasive Developmental Disorder—Not Otherwise Specified (*N* = 5), who did not carry any additional diagnosis of Fragile-X, Tuberous Sclerosis or any known neurological conditions other than epilepsy. Consistent with our prior research published with this sample (Demopoulos et al., 2023), participants between 5–18 years were included unless MEG or MRI were contraindicated due to metal in the body. Diagnostic assignment was informed by information obtained from the Autism Diagnostic Interview-Revised (ADI-R; Lord et al., 1994), the Autism Diagnostic Observation Schedule (ADOS; Lord et al., 1989), a neuropsychological history questionnaire, and relevant language and intelligence test performance. The diagnosis of ASD was confirmed by the neuropsychology team under the supervision of a licensed clinical neuropsychologist according to DSM-IV-TR criteria. Symptom criteria for ASD were contextualized by relevant language and intelligence testing performance and supported by administration of gold standard diagnostic tools, including the Autism Diagnostic Interview-Revised (ADI-R; Lord et al., 1994) and the Autism Diagnostic Observation Schedule (ADOS; Lord et al., 1989), along with information provided by the caregiver on a neuropsychological history questionnaire. All participants spoke English as a primary language, and six were multilingual. Specifically, in addition to English, one participant also spoke Russian, one spoke Tagalog, one spoke Lao, one spoke Marathi and Gujarati, and three participants spoke Spanish. Participants were not asked to refrain from taking their medications. Eleven participants were taking antidepressant medication, 12 were taking stimulants, 10 were taking antipsychotic drugs, 7 were taking anticonvulsant medications, 7 were taking antihistamines, 4 were taking sedative drugs, 2 were taking anxiolytics, two used steroid inhalers, one used a bronchodilator, one was taking a beta blocker, and two were taking cognition enhancing medications. Demographic data are presented in Table 1.

**Table 1:** Participant demographics and standardized scores.

	*M*	SD
	Range
Full scale intelligence	83.65	22.31
Quotient	46–136	
Language		
Expressive index	82.08	26.44
	45–132	
Receptive index	82.38	23.76
	45–131	
Vocabulary
Expressive	91.73	20.47
	42–145	
Receptive	87.70	27.09
	20–148	
Phonological awareness	93.13	18.27
	61–143	
Phonological memory	88.13	14.27
	52–118	
Rapid naming	86.63	18.38
	49–136	
Articulation	95.76	13.98
	40–110	
Gender (N)		
	Male	40
	Female	17
Ethnicity (N)		
	Caucasian	37
	Hispanic	6
	Asian	4
	African American	4
	Multiracial	5
	Other	1

### Procedures

Study procedures are similar to those previously reported in our study examining relations between rapid processing of puretone sounds and communication abilities in this sample (Demopoulos et al., 2023). After obtaining consent and assent, study tasks were typically completed in five visits, including three visits for diagnostic and neuropsychological testing, and two visits for MEG and MRI scans, respectively. In order to meet a range of participants’ needs, we offered breaks throughout testing, practice sessions for scans, and the opportunity to break up visits into shorter appointments.

### Measures

#### Diagnostic

The ADI-R (Lord et al., 1994) and the ADOS (Lord et al., 1989) were administered to assess for symptoms of ASD. This information was contextualized according to the participant’s language and general intellectual abilities, which were assessed via the age-appropriate Wechsler intelligence test, including the Wechsler Intelligence Scale for Children-Fourth Edition (WISC-IV; Wechsler, 2003), the Wechsler Adult Intelligence Scale-Fourth Edition (WAIS-IV; Wechsler, 2008), or the Wechsler Preschool and Primary Scale of Intelligence-Third Edition (WPPSI-III; Wechsler, 2002).

#### Verbal communication

The Clinical Evaluation of Language Fundamentals-Fourth Edition (CELF-4; Semel et al., 2003) was administered to derive norm-referenced indices of receptive and expressive language abilities. Expressive and receptive vocabulary were assessed via the Expressive Vocabulary Test (EVT; Williams, 1997) and Peabody Picture Vocabulary Test-3^rd^ Edition (PPVT-3; Dunn, 1997), respectively. The Sounds-In-Words subtest of the Goldman-Fristoe Tests of Articulation-2nd Edition (GFTA-2; Goldman and Fristoe, 2000) was administered to evaluate articulatory accuracy. Finally, phonological processing abilities were evaluated on the Comprehensive Test of Phonological Processing (CTOPP; Rashotte et al., 1999) via the phonological awareness, phonological memory, and rapid naming indices.

#### Cortical auditory processing

A MEG Rapid Speech Sound Processing Task was performed to capture auditory evoked fields in response to hearing two different speech sounds (“ga” and “da”) presented in rapid succession. This task involved three different stimulus conditions. The first captured response to single speech sounds (“ga” or “da”). The second allowed us to quantify via response two different speech sounds (“ga” and “da”). The third was designed to assess sensory gating (response to pairs of the same speech sounds); however, we determined that sensory gating could not be assessed for the many participants who demonstrated a rapid processing impairment. Specifically, these participants had impaired response to the second speech sound in the two different sounds condition. Thus, the gating response (indicated by a reduced response to the second sound in the two same sounds condition) risks being confounded with impaired rapid processing. For this reason, the focus of this study is on condition 2: response to two different speech sounds.

Data from 150 trials consisting of the randomized paired speech sounds (i.e., either “ga” follow by “da” or “da” follow by “ga”) presented 300 ms apart with an inter-trial-interval of 2,000 ms were averaged. All stimuli were presented at peak amplitude of 75 dB SPL through loudspeakers to ensure that stimuli were audible to all participants. Data were collected using a 306-channel biomagnetometer system (VectorView, Elekta, Oy, Helsinki), consisting of a planar gradiometer and magnetometer array distributed at 102 spatial positions, with one magnetometer and a pair of orthogonal planar gradiometers at each location. Prior to entering the scanner four small coils were placed on the head for digitization. A 3D-digitizer defined a head-centered coordinate frame using the nasion and peri-auricular points, as well as the position of the coils within the frame. Participants were scanned in a supine position to stabilize head position and reduce movement. During the scan, the coils were energized and localized by the sensor array to define the position of the sensors relative to the head. Because the rapid speech processing task required only passive exposure to auditory stimuli, a movie was concurrently played without sound to increase participant comfort, consistent with other studies of auditory processing in this population (Oram Cardy et al., 2008; Roberts et al., 2008, 2012; Edgar et al., 2013, 2014).

A 1,000 Hz digitization rate with a 0.1–300 Hz bandwidth was used for data collection. Signal space projections (SSP) and signal space separation with temporal extension (Taulu and Hari, 2009) were used to identify artifacts from proximal, and distal noise sources, respectively. Any epochs that contained large artifacts (> 2pT) or evidence of eye blinks or movement upon visual inspection were rejected. Single trial epochs were then generated and averaged with a baseline of 250 ms and a post-stimulus duration of 1,000 ms. Datasets in which a minimum of 130 out of the 150 trials were included. Baseline correction and 1–30 Hz band-pass filtering were performed on averaged responses. Trials ordered “ga”/“da” were averaged with trials ordered “da”/“ga” to maximize the signal to noise ratio.

A dipole fitting approach was adopted for source localization using the Neuromag Xfit program. Specifically, bilateral temporal dipole sources were placed and their positions and orientations were optimized over a 50 millisecond window spanning the peak latency of the M100 response. The dipole fit coordinates were confirmed to localize in the superior temporal perisylvian regions of the participant’s T1-weighted MRI. Left and right hemisphere responses were optimized simultaneously and a spherical head model was used. The resultant dipole model was then held fixed and source waveforms were generated by ‘passing’ the average evoked response through each individual participant’s fixed model. This method of source space projection was previously described by Tesche et al. (1995) and Wilson et al. (2008).

**Figure 1:**
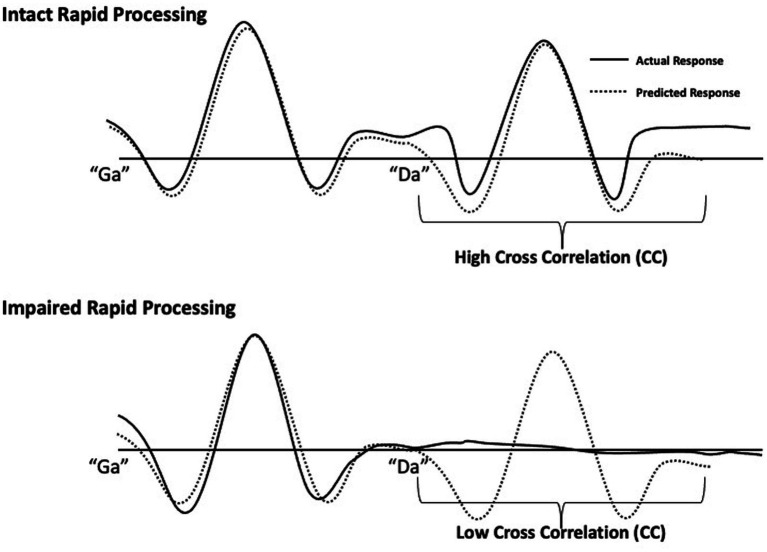
Schematic of rapid speech sound processing measurement. Individual participant responses to single speech sounds (“ga” or “da”) at 0–300 ms were projected onto the 300–600 ms time window to generate an interpolated waveform (dotted lines). This dotted line represents a predicted response to two different speech sounds presented 300 ms apart by assuming identical responses to each sound. These predicted waveforms were generated for each participant as a standard of comparison for their intact rapid processing response. For the 300–600 ms window, each participant’s predicted response to the second sound was compared to their actual response (solid lines) to the second sound by computing cross correlations. The clear response to both sounds (top drawing) demonstrates intact rapid processing and is characterized by high agreement between predicted and actual responses. The impaired rapid processing response (bottom drawing), illustrated by an absent response to the second sound, results in low agreement.

Rapid speech sound processing was determined by the response quality of the second sound (Oram-Cardy et al., 2005) in the “ga”/“da” and “da”/“ga” pairs. Specifically, a predicted waveform was created by projecting the response to the initial sound onto the 300-600 ms time window. Zero lag cross correlation coefficients (CCs) were calculated to measure agreement between a participant’s actual auditory waveform response to the two sounds and the predicted waveform. Higher values indicate greater agreement and lower values indicating poor agreement between waveforms, as described in Demopoulos et al. (2023). Intact rapid processing is characterized by a waveform with two strong responses to the first and second speech sound, respectively. Thus, when rapid auditory processing is intact, there is high agreement (reflected in a high CC value) between the actual and the predicted waveform. In contrast, poor response quality for the second sound would result in low agreement between actual and predicted waveforms (low CC value), indicating impaired rapid processing (Figure 1). CC analyses were performed in SPSS Version 20. To calculate CC values, amplitudes of the bilateral source waveforms were extracted in 5 millisecond steps and the bilateral amplitudes for the 300-600 ms time window were compared to the predicted amplitudes to yield separate CC values for right and left hemisphere responses for each participant.

#### Data analytic plan

Based on previous research reporting that rapid auditory processing of puretone sounds is directly associated with phonological awareness and that phonological awareness mediates the association between rapid puretone processing and overall language abilities in individuals with ASD (Demopoulos et al., 2023), we hypothesized that all measures of verbal communication abilities would be directly associated with MEG indices of rapid speech sound processing. These hypotheses were tested by computing Pearson correlations between bilateral MEG rapid speech processing indices and verbal communication measures, including receptive and expressive language (CELF-4), articulation (GFTA-2), receptive vocabulary (PPVT-3), expressive vocabulary (EVT), and phonological awareness, phonological memory, and rapid naming (CTOPP). Age-scaled scores based on the standardization sample were used for all measures of verbal communication to control for the effects of age. Standardization sample means and standard deviations were 100 ± 15 for all scores. Cross correlation values for the rapid speech processing index were transformed to Fisher’s Z in order to correct for the non-normality of the r distribution before being subject to further analysis.

## Results

**Figure 2:**
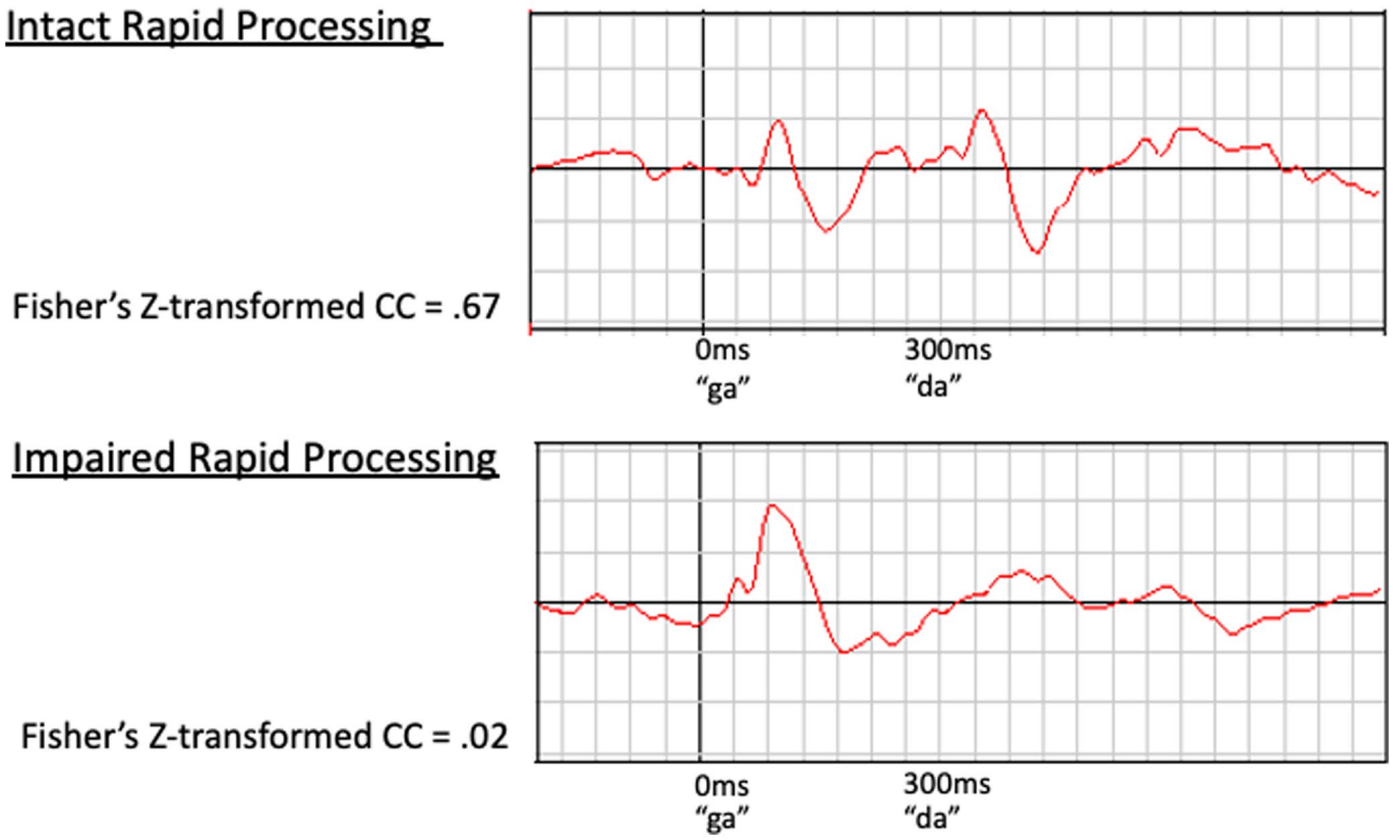
Examples of waveforms demonstrating intact (Top) and impaired (Bottom) rapid speech sound processing. The waveform on the top demonstrates a clear, timely response to both speech sounds (100 ms after stimulus presentation) in a participant with intact rapid processing. In contrast, the bottom waveform demonstrates impaired rapid processing. This participant’s initial response is present and clear at approximately 100 ms; however, the response to the second speech sound is delayed, prolonged, and poorly defined. This impaired response to the second speech sound corresponds to a low *z*-transformed CC value (*z* = 0.02) relative to the higher value (*z* = 0.67) for the intact waveform (top).

Examples of waveforms and corresponding cross correlation values for participants with intact versus impaired rapid processing are presented in Figure 2. Cortical response to the second speech sound was compared to the 300–600 ms time window of the predicted waveform, as described in Demopoulos et al. (2023). Z-transformed cross correlation values represented a range of low to high agreement (−1.02 to 1.80 for left hemisphere and –1.15 to 1.23 for right hemisphere). Thus our dataset had adequate variance in rapid processing. No significant within-participant interhemispheric differences in rapid processing were identified by paired samples t-tests, with right hemisphere *M* = 0.283, SD = 0.462 and left hemisphere *M* = 0.241, SD = 0.450, *t*(56) = −0.834, *p* = 0.408. Further, cortical rapid processing was not associated with age (*r* = −0.151, *p* = 0.263 for right hemisphere and *r* = −0.085, *p* = 0.530 for left hemisphere).

**Figure 3:**
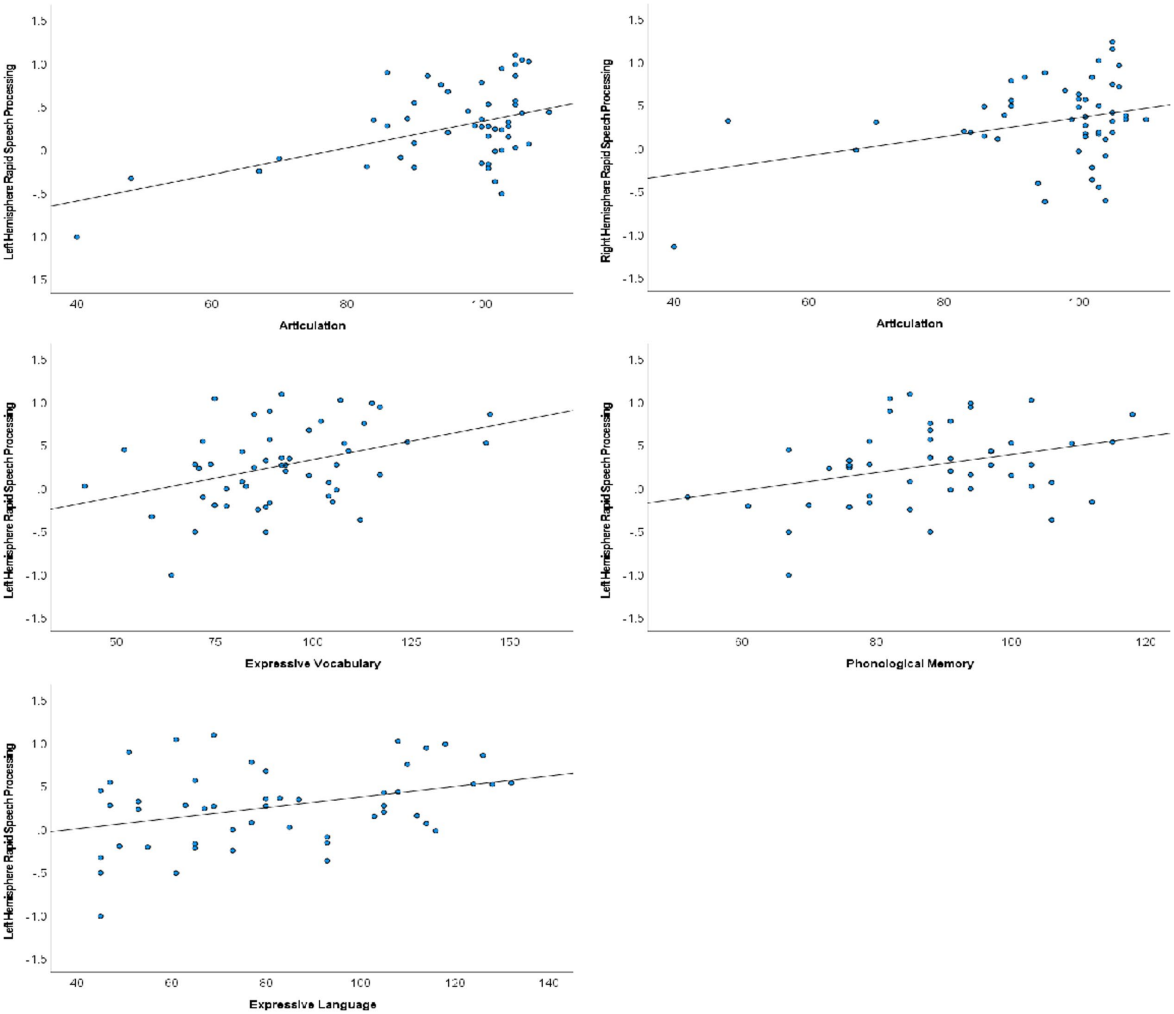
Scatterplots of rapid speech sound processing and verbal communication performance. Significant associations were identified between bilateral rapid processing and articulation, whereas associations between rapid processing and phonological memory and expressive vocabulary and language were limited to the left hemisphere.

Following Hochberg FDR correction to account for performing each hypothesized analysis twice (once in each hemisphere), rapid auditory processing of speech sounds was bilaterally associated with speech articulation (*N* = 50; LH: *r* = 0.463, *p* < 0.001; RH: *r* = 0.328, *p* = 0.020). Left hemisphere rapid processing was additionally associated with expressive language (CELF-IV ELI; *N* = 49; *r* = 0.354, *p* = 0.013), expressive vocabulary (EVT; *N* = 51; *r* = 0.384, *p* = 0.005), and phonological memory (CTOPP PM; *N* = 48; *r* = 0.325, *p* = 0.024). Scatterplots of significant correlations are presented in Figure 3. Receptive language, phonological awareness, and rapid naming were not significantly associated with cortical rapid speech sound processing in either hemisphere.

Because significant associations were identified between phonological memory and rapid processing, but not between rapid processing and receptive language, we performed *post hoc* analyses to determine if phonological memory mediated the relationship between cortical rapid speech processing and receptive language. Two mediation analyses, one for left hemisphere (LH) cortical rapid speech processing and one for right hemisphere (RH) cortical rapid speech processing, were performed using SPSS 27, consistent with methods described in Demopoulos et al. (2023). For each mediation analysis (LH and RH), two linear regression analyses were performed to estimate direct effects of (1) cortical rapid speech processing on phonological memory, and (2) rapid speech processing and phonological memory on receptive language. The Sobel Test (Sobel, 1982) was then applied to compare the unstandardized beta weight/standard error (Β/SE) for rapid speech processing in the first of these regression models (LH Β/SE = 10.156/4.351; RH Β/SE = 0.465/4.453) to the Β/SE for phonological memory in the second regression model (LH Β/SE = 1.252/0.197; RH Β/SE = 1.272/0.188). Phonological memory mediated the effect of cortical rapid speech processing on receptive language in the left (*z* = 2.191, *p* = 0.028), but not right hemisphere. The beta coefficient for rapid speech processing in the first regression model was multiplied by the beta coefficient for phonological processing in the second regression model to calculate point estimates of the mediated effects of rapid speech processing and receptive language (LH: 12.715; RH: 0.591).

## Discussion

The relationship between abnormal cortical auditory response and language impairment in individuals with ASD has been established (Port et al., 2015); however, our understanding of the process by which auditory dysfunction impacts development of verbal communication is only emerging. The present study was the first to examine specific domains of overall language function (expressive and receptive language) as well as component skills that support overall language (expressive and receptive vocabulary, articulation, phonological awareness and memory, and rapid naming) in relation to cortical rapid processing of speech sounds in children with ASD. Quality of rapid processing of speech sounds was found to be associated with both basic verbal communication and overall language skills. These findings are consistent with previous MEG studies reporting associations between rapid processing for more basic sounds and language abilities (Oram-Cardy et al., 2005; Demopoulos et al., 2023). The present study builds upon the prior work of Oram-Cardy et al. by identifying associations with the specific verbal communication skills that were related to rapid auditory processing abilities, including articulation, phonological memory, and expressive language and vocabulary. In our prior work examining associations between these verbal communication skills and rapid processing of basic puretone sounds, associations were identified between rapid processing (bilaterally) and the component skills that support language (phonological awareness, vocabulary, and articulation), but not overall expressive or receptive language skills (Demopoulos et al., 2023). Instead, phonological awareness was found to mediate the relation between rapid processing and overall language abilities.

In contrast, in the current study, associations were bilateral only for articulation, and left hemisphere rapid processing of speech sounds was associated with expressive vocabulary, phonological memory and overall expressive language. These results suggest that difficulty keeping up with processing rapid speech sounds directly impacts speech articulation and language production to a greater extent than language comprehension. Indeed, prior work in speech neuroscience has established that processing the auditory feedback self-produced speechis integral for maintaining speech accuracy (Houde et al., 2002; Houde and Jordan, 2002; Houde and Nagarajan, 2011; Chang et al., 2013; Kort et al., 2014; Martin et al., 2018). Previous work using altered feedback paradigms in an attempt to isolate the contribution of auditory feedback processing to speech motor control have demonstrated abnormal response to altered auditory feedback in speech-impaired participants with 16p11.2 deletions (Demopoulos et al., 2018), a copy number variant that is present in ~ 1% of those with ASD and is commonly associated with prominent speech deficits and developmental coordination and phonological processing disorders (Hanson et al., 2015; Fedorenko et al., 2016). These previous findings, taken together with results from the current study, may suggest that an impairment in processing the rapid auditory information produced by speech could impact one’s vocal motor control via impact on feedback processing efficiency and have a cascade impact on ability to produce speech and subsequently, develop expressive language.

Specifically, models of speech production demonstrate that online auditory monitoring of one’s own speech is utilized for ongoing vocal motor control (Houde and Jordan, 2002; Houde and Nagarajan, 2011). This auditory feedback of one’s own speech is compared against a feedforward prediction of that auditory feedback derived from the speaker’s intention. The present study identified an association between cortical rapid processing of speech sounds and phonological memory. If impairment of rapid processing of speech sounds affects ability to hold auditory information in the immediate memory, then this could be a mechanism by which a rapid processing deficit could impact online processing of auditory feedback of one’s own speech and subsequently have an adverse impact on speech production.

Further, rapid naming scores were not significantly associated with cortical rapid speech sound processing in either hemisphere. This stands to reason, as rapidly producing speech requires greater reliance on feedforward control mechanisms for speech production, as there is less time to consider auditory feedback. Thus, the association between rapid speech sound processing and untimed measures of expressive verbal communication (articulation, expressive vocabulary, and overall expressive language skills), which rely on a balance of feedforward and feedback control systems, and the absence of such an association with speeded measures of rapid speech (which are much more heavily weighted on feedforward control), further supports the interpretation that deficits in rapid auditory processing of speech sounds impacts expressive communication via impact on vocal motor control.

Receptive language was not directly associated with rapid speech processing; however, the association was mediated by phonological memory. This suggests that the impact of rapid processing impairment on ability to mentally hold and manipulate phonological information may, in turn, impact ability to process the language of others. Likewise, phonological awareness was not associated with rapid speech sound processing; however, phonological awareness has been previously associated with rapid processing of more basic puretone sounds (Demopoulos et al., 2023), suggesting that difficulty processing basic acoustic information at the rapid pace necessary for accessing the phonological structure of spoken language may impact language development as well. The present study extends upon this finding, suggesting that cortical rapid processing of phonemes is directly associated with spoken verbal communication skills and indirectly associated with receptive language skills via impact on phonological memory.

### Limitations and future directions

The present study examined associations between rapid processing of speech sounds in English speaking children and adolescents. Future research is necessary to understand the applicability of these findings and their interpretation for non-English speakers. These results focused on overall language and its component skills and did not explore nonverbal aspects of vocal communication. Future research is necessary to understand if rapid processing impairment is associated with other acoustic vocal differences that are common in autism, such as differences in rate of speech. Further, while associations were identified, the study design did not allow for conclusions regarding direction of impact, which can only be speculated. Likewise, it is not clear that these results are specific to the ASD population, as this study lacked a control group for comparison. Future studies focused on remediating rapid processing delays and using an experimental, placebo control design will be necessary to demonstrate the hypothesized interpretation of causal impact of rapid processing delays on speech and language skills in ASD. Comparison to neurotypical control group as well as individuals with other neurodevelopmental disorders in which verbal communication skill are impacted will be necessary to determine the specificity of these associations to the ASD population versus their applicability to other groups. Additionally, our mediation analyses were performed in SPSS, which does not provide goodness of fit indices to evaluate model fit. Finally, participants were not asked to discontinue medications in order to participate in the study, and it is likely that certain medications taken by participants in our sample would have an effect on brain activity in a way that would impact their cortical response to the rapidly presented speech sound stimuli in this experiment. Nevertheless, the cortical activity recorded during the MEG scan would reflect the conditions of their daily functioning (on those medications), and performance on verbal communication measures was also assessed while on medications. Thus, the potential medication effects on the variables being compared were consistent within subject. Nevertheless, inability to quantify medication effects is a limitation of this work. Future research is necessary to understand the effects of different medications on the associations reported in this study.

## Data availability statement

The raw data supporting the conclusions of this article will be made available by the authors, without undue reservation.

## Ethics statement

The studies involving humans were approved by Mind Research Network and Alexian Center for Brain Research. The studies were conducted in accordance with the local legislation and institutional requirements. Written informed consent for participation in this study was provided by the participants’ legal guardians/next of kin.

## Author contributions

CD and JL were responsible for conceptualization and design of the study. CD was additionally responsible for imaging data processing, analysis, interpretation, and writing of the manuscript. BK, NB, and KP were responsible for data acquisition and processing. SS contributed writing in the introduction. JL was the PI of this study and was responsible for the conceptualization, design, and execution of the project as well as supervision of data analysis procedures, interpretation and writing of the manuscript. All authors contributed to the article and approved the submitted version.

## Funding

This research was supported by the National Institutes of Health (grant numbers R01HD051747-01A1, K23DC016637-01A1, and R01DC019167-01A1), Autism Speaks Royal Archmasons Central Auditory Processing Disorder Awards (11637 and 13439), and UCSF Weill Institute for Neuroscience Weill Award for Clinical Neuroscience Research (2016038).

## Conflict of interest

The authors declare that the research was conducted in the absence of any commercial or financial relationships that could be construed as a potential conflict of interest.

## Publisher’s note

All claims expressed in this article are solely those of the authors and do not necessarily represent those of their affiliated organizations, or those of the publisher, the editors and the reviewers. Any product that may be evaluated in this article, or claim that may be made by its manufacturer, is not guaranteed or endorsed by the publisher.

## References

[ref1] Alcántara J. I. Weisblatt E. J. L. Moore B. C. J. Bolton P. F. (2004). Speech-in-noise perception in high-functioning individuals with autism or Asperger’s syndrome. J. Child Psychol. Psychiatry 45, 1107–1114. doi: 10.1111/j.1469-7610.2004.t01-1-00303.x 15257667

[ref2] Berman J. I. Edgar J. C. Blaskey L. Kuschner E. S. Levy S. E. Ku M.. (2016). Multimodal diffusion-MRI and MEG assessment of auditory and language system development in autism Spectrum disorder. Front. Neuroanat. 10:30. doi: 10.3389/fnana.2016.00030, PMID: 27047349 PMC4803725

[ref3] Bhatara A. Babikian T. Laugeson E. Tachdjian R. Sininger Y. S. (2013). Impaired timing and frequency discrimination in high-functioning autism spectrum disorders. J. Autism Dev. Disord. 43, 2312–2328. doi: 10.1007/s10803-013-1778-y, PMID: 23386117

[ref4] Bonnel A. Mottron L. Peretz I. Trudel M. Gallun E. Bonnel A.-M. (2003). Enhanced pitch sensitivity in individuals with autism: a signal detection analysis. J. Cogn. Neurosci. 15, 226–235. doi: 10.1162/089892903321208169 12676060

[ref5] Chang E. F. Niziolek C. A. Knight R. T. Nagarajan S. S. Houde J. F. (2013). Human cortical sensorimotor network underlying feedback control of vocal pitch. Proc. Natl. Acad. Sci. U. S. A. 110, 2653–2658. doi: 10.1073/pnas.1216827110, PMID: 23345447 PMC3574939

[ref6] Demopoulos C. Aitkens A. Findley A. Mizuiri D. Honma S. Desai S. S.. (2017). Magnetoencephalographic imaging of auditory and somatosensory cortical responses in children with autism and sensory processing dysfunction. Front. Hum. Neurosci. 11:259. doi: 10.3389/fnhum.2017.00259, PMID: 28603492 PMC5445128

[ref7] Demopoulos C. Hopkins J. Kopald B. E. B. E. Paulson K. Doyle L. Andrews W. E. W. E.. (2015). Deficits in auditory processing contribute to impairments in vocal affect recognition in autism spectrum disorders: a MEG study. Neuropsychology 29, 895–908. doi: 10.1037/neu0000209, PMID: 26011112 PMC4640958

[ref8] Demopoulos C. Kopald B. E. Bangera N. Paulson K. Lewine J. D. (2023). Rapid auditory processing of Puretones is associated with basic components of language in individuals with autism spectrum disorders. Brain Lang. 238:105229. doi: 10.1016/j.bandl.2023.105229 36753824 PMC10029928

[ref9] Demopoulos C. Kothare H. Mizuiri D. Henderson-Sabes J. Fregeau B. Tjernagel J.. (2018). Abnormal speech motor control in individuals with 16p11.2 deletions. Sci. Rep. 8:1274. doi: 10.1038/s41598-018-19751-x, PMID: 29352208 PMC5775320

[ref10] Demopoulos C. Lewine J. D. J. D. (2016). Audiometric profiles in autism spectrum disorders: does subclinical hearing loss impact communication? Autism Res. 9, 107–120. doi: 10.1002/aur.1495, PMID: 25962745 PMC4641833

[ref11] DePape A.-M. R. Hall G. B. C. Tillmann B. Trainor L. J. (2012). Auditory processing in high-functioning adolescents with autism Spectrum disorder. PLoS ONE 7:e44084. doi: 10.1371/journal.pone.0044084, PMID: 22984462 PMC3440400

[ref12] Dunn L. M. (1997). Peabody Picture Vocabulary Test-3rd Edition (PPVT-3). St. Paul, MN, American Guidance Service.

[ref13] Edgar J. C. Khan S. Y. Blaskey L. Chow V. Y. Rey M. Gaetz W.. (2013). Neuromagnetic oscillations predict evoked-response latency delays and core language deficits in autism spectrum disorders. J. Autism Dev. Disord. 45, 395–405. doi: 10.1007/s10803-013-1904-x, PMID: 23963591 PMC5012005

[ref14] Edgar J. C. Lanza M. R. Daina A. B. Monroe J. F. Khan S. Y. Blaskey L.. (2014). Missing and delayed auditory responses in young and older children with autism spectrum disorders. Front. Hum. Neurosci. 8, 1–13. doi: 10.3389/fnhum.2014.00417, PMID: 24936181 PMC4047517

[ref15] Fedorenko E. Morgan A. Murray E. Cardinaux A. Mei C. Tager-Flusberg H.. (2016). A highly penetrant form of childhood apraxia of speech due to deletion of 16p11.2. Eur. J. Hum. Genet. 24, 302–306. doi: 10.1038/ejhg.2015.149, PMID: 26173965 PMC4717199

[ref16] Foss-Feig J. H. Schauder K. B. Key A. P. Wallace M. T. Stone W. L. (2017). Audition-specific temporal processing deficits associated with language function in children with autism spectrum disorder. Autism Res. 10, 1845–1856. doi: 10.1002/aur.1820, PMID: 28632303 PMC6007978

[ref17] Gage N. M. Siegel B. Callen M. Roberts T. (2003). Cortical sound processing in children with autism disorder: an MEG investigation. Neuroreport 14, 2047–2051. doi: 10.1097/01.wnr.0000090030.46087, PMID: 14600495

[ref18] Goldman R. Fristoe M. (2000). Goldman-Fristoe Test of Articulation-2nd Edition (GFTA-2). St. Paul, MN: American Guidance Service.

[ref19] Hanson E. Bernier R. Porche K. Jackson F. I. Goin-kochel R. P. Snyder L. G.. (2015). The cognitive and behavioral phenotype of the 16p11.2 deletion in a clinically ascertained population. Biol. Psychiatry 77, 785–793. doi: 10.1016/j.biopsych.2014.04.021, PMID: 25064419 PMC5410712

[ref20] Heaton P. (2005). Interval and contour processing in autism. J. Autism Dev. Disord. 35, 787–793. doi: 10.1007/s10803-005-0024-7, PMID: 16283085

[ref21] Houde J. F. Jordan M. I. (2002). Sensorimotor adaptation of speech: compensation and adaptation. J. Speech Lang. Hear. Res. 45, 295–310. doi: 10.1044/1092-4388(2002/023) 12003512

[ref22] Houde J. F. Nagarajan S. S. (2011). Speech production as state feedback control. Front. Hum. Neurosci. 5, 1–14. doi: 10.3389/fnhum.2011.00082, PMID: 22046152 PMC3200525

[ref23] Houde J. F. Nagarajan S. S. Sekihara K. Merzenich M. M. (2002). Modulation of the auditory cortex during speech: an MEG study. J. Cogn. Neurosci. 14, 1125–1138. doi: 10.1162/089892902760807140 12495520

[ref24] Kamita M. K. Silva L. A. F. Magliaro F. C. L. Fernandes F. D. Matas C. G. (2021). Auditory event related potentials in children with autism spectrum disorder. Int. J. Pediatr. Otorhinolaryngol. 148:110826. doi: 10.1016/j.ijporl.2021.110826 34246067

[ref25] Kargas N. Lopez B. Reddy V. Morris P. López B. Reddy V.. (2015). The relationship between auditory processing and restricted, repetitive behaviors in adults with autism spectrum disorders. J. Autism Dev. Disord. 45, 658–668. doi: 10.1007/s10803-014-2219-2, PMID: 25178987

[ref27] Klin A. (1993). Auditory brainstem responses in autism: brainstem dysfunction or peripheral hearing loss? J. Autism Dev. Disord. 23, 15–35. doi: 10.1007/BF01066416 8463195

[ref28] Kort N. S. Nagarajan S. S. Houde J. F. (2014). A bilateral cortical network responds to pitch perturbations in speech feedback. NeuroImage 86, 525–535. doi: 10.1016/j.neuroimage.2013.09.042, PMID: 24076223 PMC4063684

[ref29] Lord C. Rutter M. Goode S. Heemsbergen J. Jordan H. Mawhood L.. (1989). Autism diagnostic observation schedule: a standardized observation of communicative and social behavior. J. Autism Dev. Disord. 19, 185–212. doi: 10.1007/BF02211841, PMID: 2745388

[ref30] Lord C. Rutter M. Le Couteur A. (1994). Autism diagnostic interview-revised: a revised version of a diagnostic interview for caregivers of individuals with possible pervasive developmental disorders. J. Autism Dev. Disord. 24, 659–685. doi: 10.1007/BF02172145, PMID: 7814313

[ref31] Martin C. D. Niziolek C. A. Duñabeitia J. A. Perez A. Hernandez D. Carreiras M.. (2018). Online adaptation to altered auditory feedback is predicted by auditory acuity and not by domain-general executive control resources. Front. Hum. Neurosci. 12:91. doi: 10.3389/fnhum.2018.00091, PMID: 29593516 PMC5857594

[ref32] Matsuzaki J. Ku M. Dipiero M. Chiang T. Saby J. Blaskey L.. (2020). Delayed auditory evoked responses in autism Spectrum disorder across the life span. Dev. Neurosci. 41, 223–233. doi: 10.1159/000504960, PMID: 32007990 PMC7044064

[ref33] Matsuzaki J. Kuschner E. S. Blaskey L. Bloy L. Kim M. Ku M.. (2019). Abnormal auditory mismatch fields are associated with communication impairment in both verbal and minimally verbal/nonverbal children who have autism spectrum disorder. Autism Res. 12, 1225–1235. doi: 10.1002/aur.2136, PMID: 31136103 PMC6710834

[ref34] Mayer J. L. Hannent I. Heaton P. F. (2014). Mapping the developmental trajectory and correlates of enhanced pitch perception on speech processing in adults with ASD. J. Autism Dev. Disord. 46, 1562–1573. doi: 10.1007/s10803-014-2207-6, PMID: 25106823

[ref35] O’Riordan M. Passetti F. (2006). Discrimination in autism within different sensory modalities. J. Autism Dev. Disord. 36, 665–675. doi: 10.1007/s10803-006-0106-1, PMID: 16639532

[ref36] Oram Cardy J. E. Ferrari P. Flagg E. J. Roberts W. Roberts T. P. L. (2004). Prominence of M50 auditory evoked response over M100 in childhood and autism. Neuroreport 15, 1867–1870. doi: 10.1097/00001756-200408260-00006, PMID: 15305126

[ref37] Oram Cardy J. E. Flagg E. J. Roberts W. Roberts T. P. L. (2005). Delayed mismatch field for speech and non-speech sounds in children with autism. Neuroreport 16, 521–525. doi: 10.1097/00001756-200504040-00021, PMID: 15770164

[ref38] Oram Cardy J. E. Flagg E. J. Roberts W. Roberts T. P. L. (2008). Auditory evoked fields predict language ability and impairment in children. Int. J. Psychophysiol. 68, 170–175. doi: 10.1016/j.ijpsycho.2007.10.015, PMID: 18304666

[ref39] Oram-Cardy J. E. Flagg C. A. E. J. Roberts W. Brian J. Roberts T. P. L. (2005). Magnetoencephalography identifies rapid temporal processing deficit in autism and language impairment. Neuroreport 16, 329–332. doi: 10.1097/00001756-200503150-00005, PMID: 15729132

[ref40] Port R. G. Anwar A. R. Ku M. Carlson G. C. Siegel S. J. Roberts T. P. L. (2015). Prospective MEG biomarkers in ASD: pre-clinical evidence and clinical promise of electrophysiological signatures. Yale J. Biol. Med. 88, 25–36. PMID: 25745372 PMC4345535

[ref41] Rashotte C. Torgesen J. Wagner R. (1999). Comprehensive Test of Phonological Processing (CTOPP). Austin, TX: ProEd.

[ref42] Rescorla L. Alley A. (2001). Validation of the language development survey (LDS). J. Speech Lang. Hear. Res. 44, 434–445. doi: 10.1044/1092-4388(2001/035), PMID: 11324663

[ref43] Riva V. Cantiani C. Mornati G. Gallo M. Villa L. Mani E.. (2018). Distinct ERP profiles for auditory processing in infants at-risk for autism and language impairment. Sci. Rep. 8:715. doi: 10.1038/S41598-017-19009-Y, PMID: 29335488 PMC5768787

[ref44] Roberts T. P. L. Cannon K. M. Tavabi K. Blaskey L. Khan S. Y. Monroe J. F.. (2011). Auditory magnetic mismatch field latency: a biomarker for language impairment in autism. Biol. Psychiatry 70, 263–269. doi: 10.1016/j.biopsych.2011.01.015, PMID: 21392733 PMC3134608

[ref45] Roberts T. P. L. Heiken K. Kahn S. Y. Qasmieh S. Blaskey L. Solot C.. (2012). Delayed magnetic mismatch negativity field, but not auditory M100 response, in specific language impairment. Neuroreport 23, 463–468. doi: 10.1097/WNR.0b013e32835202b6, PMID: 22551948 PMC3345126

[ref46] Roberts T. P. L. Khan S. Y. Rey M. Monroe J. F. Cannon K. Woldoff S.. (2010). MEG detection of delayed auditory evoked responses in autism Spectrum disorders: towards an imaging biomarker for autism. Autism Res. 3, 8–18. doi: 10.1002/aur.111.MEG, PMID: 20063319 PMC3099241

[ref47] Roberts T. P. L. Kuschner E. S. Edgar J. C. (2021). Biomarkers for autism spectrum disorder: opportunities for magnetoencephalography (MEG). J. Neurodev. Disord. 13, 34–39. doi: 10.1186/s11689-021-09385-y, PMID: 34525943 PMC8442415

[ref48] Roberts T. P. L. Lanza M. R. Dell J. Qasmieh S. Hines K. Blaskey L.. (2013). Maturational differences in thalamocortical white matter microstructure and auditory evoked response latencies in autism spectrum disorders. Brain Res. 1537, 79–85. doi: 10.1016/j.brainres.2013.09.011, PMID: 24055954 PMC3970268

[ref49] Roberts T. P. L. Matsuzaki J. Blaskey L. Bloy L. Edgar J. C. Kim M.. (2019). Delayed M50/M100 evoked response component latency in minimally verbal/nonverbal children who have autism spectrum disorder. Mol. Autism. 10, 34–11. doi: 10.1186/s13229-019-0283-3, PMID: 31428297 PMC6694560

[ref50] Roberts T. P. L. Schmidt G. L. Egeth M. Blaskey L. Rey M. M. Edgar J. C.. (2008). Electrophysiological signatures: magnetoencephalographic studies of the neural correlates of language impairment in autism spectrum disorders. Int. J. Psychophysiol. 68, 149–160. doi: 10.1016/j.ijpsycho.2008.01.012, PMID: 18336941 PMC2397446

[ref51] Russo N. Nicol T. Trommer B. Zecker S. Kraus N. (2009). Brainstem transcription of speech is disrupted in children with autism spectrum disorders. Dev. Sci. 12, 557–567. doi: 10.1111/j.1467-7687.2008.00790.x, PMID: 19635083 PMC2718770

[ref52] Schmidt G. L. Rey M. M. Oram Cardy J. E. Roberts T. P. L. (2009). Absence of M100 source asymmetry in autism associated with language functioning. Neuroreport 20, 1037–1041. doi: 10.1097/WNR.0b013e32832e0ca7, PMID: 19491710 PMC2795634

[ref53] Semel E. Wiig E. Secord W. (2003). Clinical Evaluation of Language Fundamentals-fourth Edition (CELF-4) The Psychological Corporation.

[ref54] Sobel M. E. (1982). “Asymptotic intervals for indirect effects in structural equations models” in Sociological Methodology. ed. Leinhart S. (Hoboken, NJ: Jossey-Bass), 290–312.

[ref55] Sparrow S. S. Cicchetti D. Balla D. (2005). Vineland Adaptive Behavior Scales, 2. Pearson Assessments.

[ref56] Sparrow S. Cicchetti D. Saulnier C. (2016). Vineland Adaptive Behavior Scales-third Edition. San Antonio, TX: Pearson Assessments.

[ref57] Stewart M. E. Griffiths T. D. Grube M. (2015). Autistic traits and enhanced perceptual representation of pitch and time. J. Autism Dev. Disord. 48, 1350–1358. doi: 10.1007/s10803-015-2517-3, PMID: 26189179

[ref58] Tas A. Yagiz R. Tas M. Esme M. Uzun C. Karasalihoglu A. R. (2007). Evaluation of hearing in children with autism by using TEOAE and ABR. Autism 11, 73–79. doi: 10.1177/1362361307070908 17175575

[ref59] Taulu S. Hari R. (2009). Removal of magnetoencephalographic artifacts with temporal signal-space separation: demonstration with single-trial auditory-evoked responses. Hum. Brain Mapp. 30, 1524–1534. doi: 10.1002/hbm.20627, PMID: 18661502 PMC6871056

[ref60] Tesche C. D. Uusitalo M. A. Ilmoniemi R. J. Huotilainen M. Kajola M. Salonen O. (1995). Signal-space projections of MEG data characterize both distributed and well-localized neuronal sources. Electroencephalogr. Clin. Neurophysiol. 95, 189–200. doi: 10.1016/0013-4694(95)00064-6, PMID: 7555909

[ref61] Tomchek S. D. Huebner R. A. Dunn W. (2014). Patterns of sensory processing in children with an autism spectrum disorder. Res. Autism Spectr. Disord. 8, 1214–1224. doi: 10.1016/j.rasd.2014.06.006

[ref62] Wechsler D. (2002). Wechsler Preschool and Primary Scale of Intelligence-third Edition (WPPSI-III). San Antonio, TX: Pearson Assessments.

[ref63] Wechsler D. (2003). Wechsler Intelligence Scale For Children-fourth Edition (WISC-IV). San Antonio, TX: Pearson Assessments.

[ref64] Wechsler D. (2008). Wechsler Adult Intelligence Scale-fourth Edition (WAIS-IV). San Antonio, TX: Pearson Assessments.

[ref65] Williams K. T. (1997). Expressive Vocabulary Test (EVT). Circle Pines, MN: American Guidance Service.

[ref66] Wilson T. W. Hernandez O. O. Asherin R. M. Teale P. D. Reite M. L. Rojas D. C. (2008). Cortical gamma generators suggest abnormal auditory circuitry in early-onset psychosis. Cerebral Cortex (New York, NY) 18, 371–378. doi: 10.1093/cercor/bhm062, PMID: 17557901 PMC2648842

